# Enhanced Recovery After Surgery (ERAS) in Latin America and the Caribbean: A Scoping Review of Implementation Strategies, Clinical Outcomes, and Health System Impact

**DOI:** 10.1002/wjs.70325

**Published:** 2026-03-16

**Authors:** Lucas Ferreira Gomes Pereira, José Eduardo Guimarães Pereira, Vinicius Caldeira Quintão, Luiz Fernando dos Reis Falcão, Bruno Araújo Borges, Bruce Biccard, Carlos Darcy Alves Bersot

**Affiliations:** ^1^ Discipline of Anesthesiology Hospital das Clínicas Faculty of Medicine University of São Paulo (FMUSP) São Paulo Brazil; ^2^ Department of Anesthesiology Hospital Unimed Volta Redonda Rio de Janeiro Brazil; ^3^ Department of Anesthesiology Pain and Critical Care Medicine Escola Paulista de Medicina—Universidade Federal de São Paulo (EPM‐UNIFESP) São Paulo Brazil; ^4^ Department of Anesthesiology Hospital DF Star Brasília Brazil; ^5^ Department of Anaesthesia and Perioperative Medicine Groote Schuur Hospital University of Cape Town Cape Town South Africa; ^6^ Department of Anesthesiology Santa Casa de Misericórdia de Campos dos Goytacazes Rio de Janeiro Brazil

**Keywords:** Caribbean, enhanced recovery after surgery, fast‐track surgery, health systems, Latin America, perioperative care

## Abstract

**Background:**

Enhanced Recovery After Surgery (ERAS) programs are increasingly recognized as effective pathways to improve perioperative outcomes, yet their implementation across Latin America and the Caribbean remains poorly mapped. Understanding current strategies, clinical results, and economic implications is essential to identify regional gaps and guide evidence‐based surgical improvement.

**Methods:**

This scoping review followed Joanna Briggs Institute (JBI) methodology and PRISMA‐ScR reporting guidelines. Searches were conducted in MEDLINE/PubMed, Web of Science, LILACS, and CENTRAL, complemented by gray literature and reference screening. Eligible studies were investigations evaluating perioperative optimization interventions in any surgical population within Latin America and the Caribbean. Data extraction included study characteristics, ERAS components, implementation strategies, clinical outcomes, and economic impact.

**Results:**

Forty‐five studies published between 2006 and 2025 were included, predominantly from Brazil (*n* = 28), Mexico (*n* = 9), Argentina (*n* = 4), and Chile (*n* = 3). Most studies implemented multimodal perioperative pathways, with the most frequent ERAS strategies being preoperative fasting abbreviation (*n* = 26), early refeeding (*n* = 22), early mobilization (*n* = 22), opioid‐sparing anesthesia (*n* = 19), preoperative education (*n* = 16), and restrictive intravenous fluids (*n* = 16). Clinical outcomes consistently demonstrated reductions in postoperative length of stay and complications. Only five studies reported economic data, all focused on hospital‐level costs, showing decreased expenditures primarily driven by shorter hospitalization.

**Conclusions:**

ERAS initiatives are increasing across Latin America and the Caribbean, with evidence suggesting reductions in hospital stay and costs. Nevertheless, adoption remains uneven and concentrated in a few countries. Expanding implementation will require addressing structural disparities and generating stronger economic and implementation‐focused evidence to support broader regional uptake.

## Graphical abstract

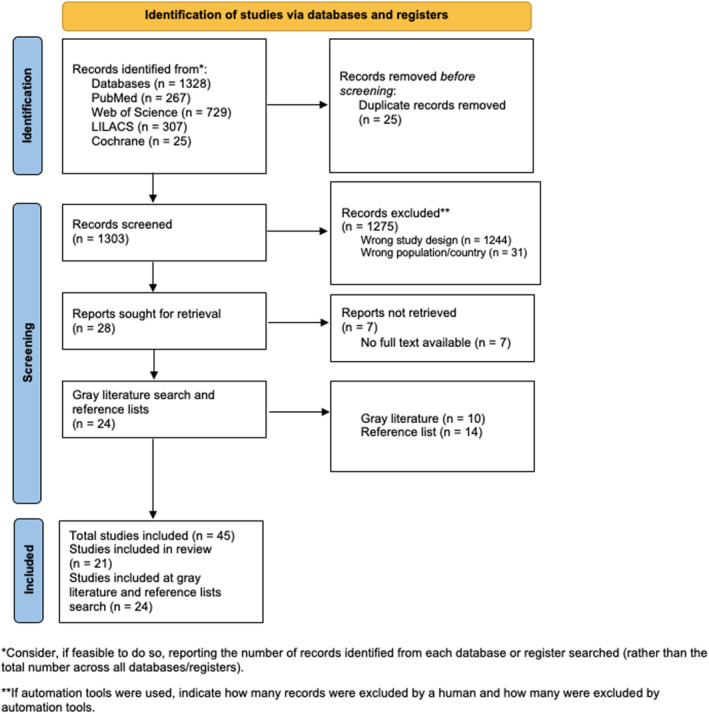

## 1 Introduction

The concept of accelerating postoperative recovery first emerged in the United States under the framework of “fast‐track” surgery, initially applied to cardiac procedures with the goal of promoting faster patient recovery [1]. This idea was later expanded by Kehlet and colleagues, who introduced a multimodal rehabilitation pathway in colorectal surgery that demonstrated significant reductions in hospital stay [2]. The Enhanced Recovery After Surgery (ERAS) programs, developed in Europe and globally disseminated, provide an evidence‐based, standardized model for perioperative care. Despite widespread adoption of several protocols in North America and Europe, their implementation in Latin America remains underreported, highlighting the need for further investigation in the region.

In Latin America, one of the first structured ERAS‐inspired initiatives was the ACERTO project in Brazil, led by Dr. Aguilar at the *Hospital Universitário Júlio Muller* (HUJM) 15 years ago, focusing on enhanced recovery after abdominal surgery [3]. ACERTO has become a regional reference, promoting ERAS principles through annual courses and seminars. While supported by the ERAS Society, the Society's formal presence in Latin America began with the ERAS Implementation Program at *St. Mark's Hospital in London* in collaboration with the *Hospital Italiano de Buenos Aires*, which was designated the first ERAS Society Center of Excellence in the region in 2014, serving as a hub for training and further implementation [4, 5].

Across high‐income countries, ERAS has become a standard of perioperative care, supported by specialty‐specific guidelines and continuous education from the ERAS Society. Evidence shows consistent benefits, including fewer complications and shorter hospital stays. In Latin America, however, implementation remains uneven, often limited to small‐scale initiatives and challenged by resource constraints, infrastructure variability, and gaps in training. Although not specific to ERAS, the LASOS study evaluated surgical patients across Latin America and revealed that, despite complication rates being similar to those in high‐income countries, mortality after complications was higher. This finding reflects deficiencies in postoperative care, prolonged hospitalizations, and limited critical care resources. These disparities highlight the need to map ERAS initiatives, identify barriers and facilitators, and develop strategies adapted to the diverse health‐care systems of the region [6, 7, 8, 9].

This scoping review aims to map and summarize the available evidence on Enhanced Recovery After Surgery (ERAS) in Latin America and the Caribbean, with a focus on describing current practices, implementation challenges, reported outcomes, and gaps in the literature. The research questions guiding this review are:

1. What research has been conducted on Enhanced Recovery After Surgery (ERAS) in Latin America and the Caribbean?
2. What are the economic impact of the ERAS implementation?
3. Which ERAS protocol recommendations have been most frequently studied in Latin America and the Caribbean?
4. What outcomes have been most frequently studied in this context?
5. What gaps remain in the literature on ERAS in Latin America and the Caribbean?

## 2 Methods

### 2.1 Study Design and Protocol Registration

This scoping review was conducted in accordance with the Joanna Briggs Institute (JBI) methodology for scoping reviews and informed by the Cochrane Handbook for Intervention Reviews [10]. Reporting followed the Preferred Reporting Items for Systematic Reviews and Meta‐Analyses extension for Scoping Reviews (PRISMA‐ScR) [11]. The review protocol was prospectively registered on the Open Science Framework (https://osf.io/9x7by/).

### 2.2 Search Strategy

The literature search was conducted using PubMed (MEDLINE), Web of Science, LILACS and the Cochrane Controlled Register of Trials (CENTRAL). We also searched for unpublished studies and gray literature through manual searches of reference lists of included articles. The initial search was performed in August 2025, followed by an updated search in September 2025. Search strategies combined MeSH terms and their synonyms adapted for each database, including “Enhanced Recovery After Surgery”, “Latin America”, “Caribbean”, “Perioperative care” as well as individual countries names in the region. No restrictions were applied regarding language or publication date.

### 2.3 Eligibility Criteria

Randomized controlled trials and observational studies conducted in Latin America and the Caribbean that evaluated the implementation of ERAS strategies were considered eligible. Studies involving patients of any age undergoing surgical procedures were included, with no restrictions regarding the type of surgery. The intervention group was defined as those receiving any perioperative measure aimed at optimizing and accelerating recovery, while the control group consisted of patients managed with standard care. Terminologies accepted for inclusion comprised “Fast‐track,” “Enhanced Recovery After Surgery (ERAS),” or other structured optimization protocols such as ACERTO.

### 2.4 Study Selection

Three reviewers (LFGP, CDAB, JEGP) imported all references into Rayyan software (Qatar Computing Research Institute, Doha, Qatar) [12] and independently screened titles and abstracts following a standardized protocol. Full texts of potentially relevant studies were then retrieved and assessed for eligibility by consensus. Any disagreements were resolved through discussion or, when necessary, by consulting additional reviewers (LFRF, VCQ). Manual searches of reference lists from included studies and relevant reviews were also performed to identify additional eligible publications.

### 2.5 Data Extraction

Two independent reviewers (LFGP and CDAB) used a standardized data extraction tool, developed by the review team, to collect information from the studies included in the scoping review. Extracted data comprised publication year, study population, country, language, type of study, ERAS components assessed, implementation strategies, reported challenges, and outcomes relevant to the review question [13]. Discrepancies between reviewers were resolved through discussion with a third reviewer (JEGP). When necessary, study authors were contacted via the corresponding email to obtain missing information.

### 2.6 Data Analysis and Presentation

The information extracted from the included studies was organized into 02 tables, emphasizing the primary data with a focus on perioperative optimization interventions, including the strategies employed, challenges encountered during implementation, and the resulting outcomes and impacts. Data were systematically charted, and the tables included the following elements: year of publication, country, journal, surgical procedures, type of study, perioperative period studied, ERAS strategy, clinical outcomes and economical impact.

## 3 Results

### 3.1 Search

**FIGURE 1:**
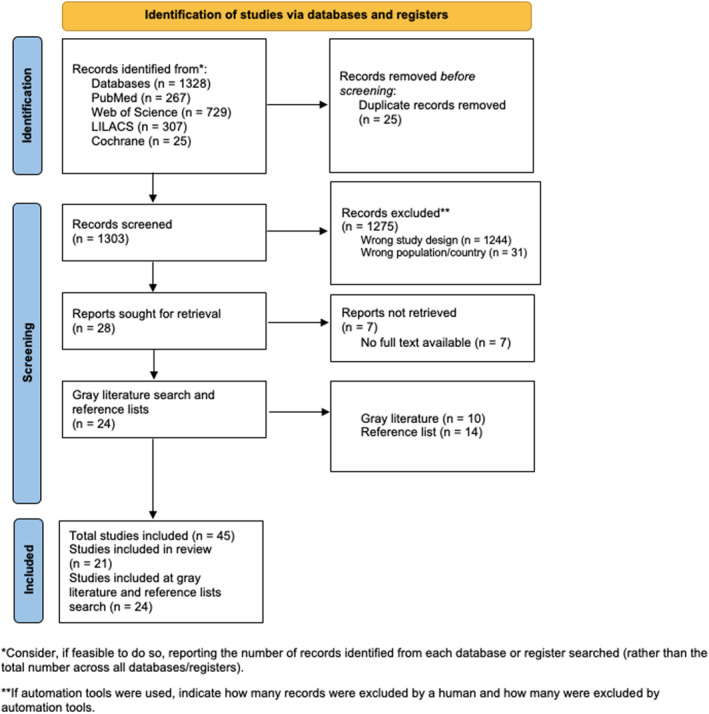
Preferred Reporting Items for Systematic Reviews and Meta‐Analysis (PRISMA) flow diagram.

The database search retrieved 1328 records, of which 25 were duplicates and subsequently removed. The remaining 1303 titles and abstracts were screened, leading to the exclusion of 1275 articles—1244 due to unsuitable study design and 31 because the population or setting was outside Latin America and Caribbean. Twenty‐eight full texts were then reviewed for eligibility, however seven could not be obtained as they were published only in conference proceedings without full‐text availability. An additional 24 records were identified through gray literature sources (*n* = 10) and reference list screening (*n* = 14). In total, 45 studies were incorporated [14, 15, 16, 17, 18, 19, 20, 21, 22, 23, 24, 25, 26, 27, 28, 29, 30, 31, 32, 33, 34, 35, 36, 37, 38, 39, 40, 41, 42, 43, 44, 45, 46, 47, 48, 49, 50, 51, 52, 53, 54, 55, 56, 57, 58], with 21 meeting the predefined inclusion criteria. The overall selection pathway is depicted in the PRISMA flow diagram (Figure 1).

### 3.2 Characteristics of Included Studies

**FIGURE 2:**
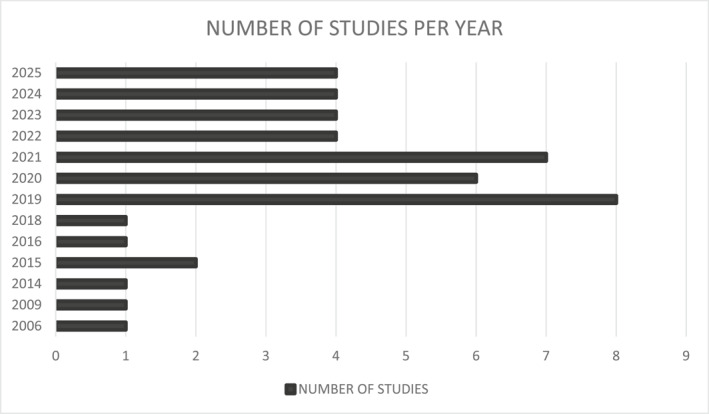
Number of studies per year.

**FIGURE 3:**
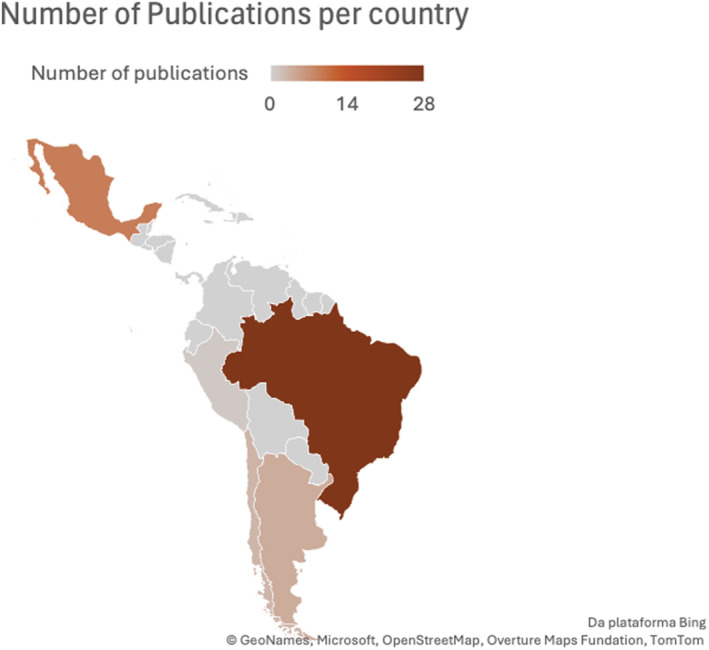
Number of publications per country.

The 45 selected studies were published between 2006 [14] and 2025 [55, 56, 57, 58] (Figure 2). The publications were from six Latin America countries: Brazil (*n* = 28); Mexico (*n* = 9); Argentina (*n* = 4); Chile (*n* = 3); Peru (*n* = 1) (Figure 3). The types of surgical procedures includes elective abdominal surgeries [14, 43, 58], large bowel surgeries [15], femur fracture surgeries [16], laparoscopic cholecistectomy [17, 46], bariatric surgery [18, 22, 35, 39], hip arthroplasty [19, 38, 53], laparoscopic appendectomy [20, 36], pediatric bowel anastomoses [21], liver surgeries [23, 28, 42], colorectal surgeries [24, 27, 29, 33, 41, 52, 54], gynecological surgeries [25, 30, 31, 48], head and neck cancer [32], brain tumor surgery [34], orthognatic surgery [37], cardiac surgery [40], knee arthroplasty [45], breast reconstruction surgery [47], cesarean section [49], pediatric colostomy closure [50], emergency laparotomy [51], laparoscopic kidney donor nephrectomy [57] (Table 1).

**TABLE 1:** Characteristics of the included studies.

Authors	Year of publication	Country	Journal	Surgical procedures	Type of study
Aguilar‐Nascimento [14]	2006	Brazil	Revista do Colégio Brasileiro dos Cirurgiões	Elective abdominal surgeries	Prospective cohort
Espindola [15]	2009	Chile	Revista chilena de cirurgia	Large bowel surgeries	Quasi—experimental study with historical controls
Imbelloni [16]	2014	Brazil	Revista do Colégio Brasileiro dos Cirurgiões	Femur fracture surgery	Quasi—experimental study
Ravanini [17]	2015	Brazil	Nutrición Hospitalaria	Laparoscopic cholecystectomy	Randomized controlled trial
Pimenta [18]	2015	Brazil	Obesity surgery	Bariatric surgery	Randomized pilot trial
Alito [19]	2016	Brazil	Nutrition journal	Hip arthroplasty	Randomized pilot trial
Trejo‐Ávila [20]	2018	Mexico	Surgical endoscopy	Laparoscopic appendectomy	Randomized controlled trial
Santos‐Jasso [21]	2019	Mexico	Journal of pediatric surgery	Pediatric elective bowel anastomoses	Randomized controlled Noninferiority trial
Gálvez‐Gallo [22]	2019	Mexico	Cirurgia y Cirurjanos	Bariatric surgery	Prospective cohort
Teixeira [23]	2019	Brazil	Brazilian archives of digestive surgery	Liver surgeries	Quasi—experimental study with historical controls
Bicudo‐Salomão [24]	2019	Brazil	Brazilian archives of digestive surgery	Colorectal surgery	Prospective cohort
Uyeda [25]	2019	Brazil	Taiwanese journal of obstetrics & gynecology	Gynecological surgeries	Quasi—experimental study with historical controls
Teixeira [26]	2019	Brazil	Brazilian archives of digestive surgery	Colorectal surgery	Quasi—experimental study with historical controls
Mc Loughlin [27]	2019	Argentina	Clinical nutrition ESPEN	Colorectal surgery	Quasi—experimental study with historical controls
Nari [28]	2019	Argentina	Cirurgia y Cirurjanos	Hepatectomy	Comparative prospective cohort study
Santiago [29]	2020	Argentina	Revista de la Facultad de Ciencias Médicas de Córdoba	Laparoscopic colorectal surgery	Prospective observational series
Marquini [30]	2020	Brazil	Revista Brasileira de ginecologia e obstetricia	Gynecological surgeries	Randomized controlled trial
Marquini [31]	2020	Brazil	Nutrition journal	Gynecological surgeries	Randomized controlled trial
de Carvalho [32]	2020	Brazil	Nutrition in clinical practice	Head and neck cancer	Randomized controlled trial
Checa [33]	2020	Peru	Revista de gastroenterología del Perú	Colorectal surgeries	Quasi—experimental study with historical controls
Neville [34]	2020	Brazil	BMC surgery	Brain tumor surgery	Quasi—experimental study with historical controls
Zandomenico [35]	2021	Brazil	Brazilian journal of anesthesiology	Bariatric surgery	Quasi—experimental study with historical controls
Núñez‐Venzor [36]	2021	Mexico	SN Comprehensive clinical Medicine	Laparoscopic appendectomy	Randomized controlled trial
Oliveira [37]	2021	Brazil	Brazilian oral research	Orthognathic surgery	Randomized controlled trial
Almeida [38]	2021	Brazil	Journal of orthopedic surgery and research	Hip arthroplasty	Quasi—experimental study with historical controls
Oliveira [39]	2021	Brazil	Obesity surgery	Bariatric surgery	Retrospective cohort study
Mejia [40]	2021	Brazil	Scientific reports	Cardiac surgery	Prospective cohort
Martínez‐Mardones [41]	2021	Chile	Revista médica de Chile	Colorectal surgery	Prospective cohort
Nari [42]	2021	Argentina	Brazilian archives of digestive surgery	Liver resection	Comparative prospective cohort study
Aguilar‐Nascimento [43]	2022	Brazil	Brazilian archives of digestive surgery	Major elective gastrointestinal procedures	Quasi—experimental study with historical controls
Stahlschmidt [44]	2022	Brazil	Anesthesia	High risk surgery patients	Quasi—experimental study with historical controls
Herros‐García [45]	2022	Mexico	Acta ortopédica Mexicana	Knee arthroplasty	Randomized controlled trial
Mendoza‐Vélez [46]	2022	Mexico	Cirurgia y Cirurjanos	Laparoscopic cholecystectomy	Prospective cohort
Gruber [47]	2023	Brazil	Brazilian journal of plastic surgery	Breast reconstruction plastic surgery	Quasi—experimental study with historical controls
Marra [48]	2023	Brazil	Revista Brasileira de ginecologia e obstetricia	Gynecological surgeries	Prospective before—and—after educational study
Pineyro [49]	2023	Mexico	Ginekologia Polska	Cesarean section	Comparative retrospective cohort study
Fernandez‐Portilla [50]	2023	Mexico	Journal of pediatric surgery	Pediatric colostomy closure	Randomized controlled trial
Fonseca [51]	2024	Brazil	European journal of trauma and emergency surgery	Emergency laparotomy	Case—matched before—and—after cohort study
Frizon [52]	2024	Brazil	Brazilian archives of digestive surgery	Colorectal surgery	Retrospective cohort study
Lima [53]	2024	Brazil	Revista Brasileira de ortopedia	Hip arthroplasty	Prospective cohort study
Riquoir [54]	2024	Chile	Updates in surgery	Colon ressections	Quasi—experimental study with historical controls
Arruda [55]	2025	Brazil	Revista do Colégio Brasileiro dos Cirurgiões	Surgical procedures	Comparative retrospective cohort study
Gonçalves [56]	2025	Brazil	Revista do Colégio Brasileiro dos Cirurgiões	Elective surgeries	Prospective before—and—after cohort study
Hernandez‐Gaytán [57]	2025	Mexico	International urology and nephrology	Laparoscopic kidney donor nephrectomy	Prospective comparative cohort study
Magro [58]	2025	Brazil	Arquivos de gastroenterologia	Gastrointestinal surgery	Comparative retrospective cohort study

### 3.3 Enhanced Recovery Strategies, Clinical Outcomes Studied and Economical Impact

This section details the specific Enhanced Recovery After Surgery (ERAS) components implemented across the studies included in this scoping review (Table 2). Most of the studies reviewed focused on the entire perioperative course, 34 studies implemented and evaluated ERAS components comprehending all the phases. A minority of the research focused on single‐phase interventions: 4 studies concentrated exclusively on preoperative components, and only 1 study focused solely on postoperative interventions.

**TABLE 2:** Enhanced recovery strategies, clinical outcomes studied and economical impact.

Authors	ERAS strategy	Clinical outcomes	Economical impact
Aguilar‐Nascimento [14]	Preoperative fasting abbreviation; Early refeeding; Goal‐directed intravenous hydration; No bowel preparation; Do not use drains or urinary catheters; Early mobilization.	No difference was found between groups in drain or catheter placement, but outcomes improved for fasting duration, oral feeding reintroduction, hospital stay, and postoperative complications.	—
Espindola [15]	Mechanical preparation only for left colon and rectal surgeries. Continuous epidural anesthesia, maintenance of normothermia, local infiltration, small incision, no routine use of catheters, reduced opioid consumption. Early mobilization, epidural catheter removed 48 h after surgery, restriction of intravenous hydration.	Reduction of hospital length stay, less postoperative pain (VAS < 2), best patient satisfaction, more postoperative hypotension, less postoperative ileum, no differences in the percentage of anastomotic dehiscence and operative mortality compared to traditional surgery.	—
Imbelloni [16]	Preoperative fasting abbreviation; Early refeeding; No rotine use of urinary catheters; Anesthetic block and opioid sparing strategies;	Preoperative nutritional assessment and fasting time; Preoperative Mean hospital length of stay; Preoperative intravenous hydration, blood replacement and hypotension; Preoperative time for early refeeding; Preoperative there was no control group for comparison.	—
Ravanini [17]	Preoperative fasting abbreviation—200 mL of carbohydrate and protein solution 2h before the procedure	PONV—1 patient (control) × 2 patients (intervention) Pain (VAS average)—0.64 (control) × 0.72 (intervention); Length of hospital stay (days)—2.05 (control) × 2.04 (intervention) Serum glucose, insulin, interleukin‐1 (IL‐1), and tumor necrosis factor‐alpha (TNF‐α);	—
Pimenta [18]	Intravenous fluids restriction; Preoperative fasting abbreviation; Early mobilization; Early refeeding; No rotine use of urinary catheters;	PONV—5 patients (control) × 2 patients (intervention) Hospital length of stay (average)—3 days (control) and 2 days (intervention); Assess the levels of glucose, insulin, C‐ reactive protein (CRP), albumin, prealbumin, alpha 1 acid glycoprotein, and interleukin 6.	—
Alito [19]	Preoperative fasting abbreviation; Preoperative supplement; Don't use drains and catheters. Restrictive intravenous fluids; Postoperative early refeeding; Early mobilization.	Hospital length of stay (median)—6 days (control) × 3 days (intervention) CRP levels on the second PO day (mg/L)—80.6 ± 10.9 (control) × 66.5 ± 16.5 (intervention)	—
Trejo‐Ávila [20]	Opioid sparing anesthesia; Don't use drains or urinary catheters; Restrictive intravenous fluids; Early mobilization and refeeding;	Postoperative pain (VAS, %)—Mild—72% (intervention) × 37.9% (control)/Moderate‐severe—28% (intervention) × 62.1% (control); Time to resume diet (minutes)—110 min (intervention) × 360 min (control); Postoperative LOS (hours—mean)—9.7 h (intervention) × 23,2 h (control);	—
Santos‐Jasso [21]	No mechanical bowel preparation; Postoperative early refeeding; Don't use rotine nasogastric tube Opioid sparing anesthesia.	Postoperative length of stay (mean, days)—1 (intervention) × 6.57 (control); Time to tolerance of ≥ 80% of regular diet, postoperatively (hours, mean)—21.7 h (intervention) × 99,8 h (control); Time to first postoperative bowel movement (hours, mean)—16.8 h (intervention) × 35,7 h (control).	—
Gálvez‐Gallo [22]	Preoperative education; Restrictive intravenous fluids; Opioid sparing anesthesia; Don't use drains or urinary catheters; Early mobilization; Early refeeding; Postoperative Breathing exercises;	Length of hospital stay (days, mean ± SD)—1.14 ± 0.9; Adherence to ERABS recommended actions;	—
Teixeira [23]	Preoperative fasting abbreviation; Preoperative education; Opioid sparing anesthesia; Early mobilization; Early refeeding;	Length of hospital stay (median—days)—5 days (intervention) × 7 days (control); Early enteral refeeding (%)—91.4% (intervention) × 50% (control);	—
Bicudo‐Salomão [24]	Preoperative fasting abbreviation; Early refeeding; Restrictive intravenous fluids; No routine mechanical bowel preparation; Early mobilization; Don't use drains or nasogastric tubes;	Preoperative fasting ≤ 4 h (%)—73.1%; Postoperative feeding ≤ 24 h (%)—96.2%; Length of stay (days, median)—9 days; Pneumonia/atelectasis (%)—26.9%;	—
Uyeda [25]	Preoperative fasting abbreviation; Opioid sparing anestesia; Don't use routine drains or nasogastric tubes; Early mobilization; Restrictive intravenous fluids;	PONV at the immediate postoperative period (%) −14.29% (control) × 4.74% (intervention); Length of hospital stay (median, hours) ‐ 52.85 h (control) × 51,65 h; Postoperative complications (%)—2% (control) × 0.5% (intervention)	—
Teixeira [26]	Preoperative education; No routine mechanical bowel preparation; Preoperative fasting abbreviation; Restrictive intravenous fluids administration; Opioid sparing anesthesia; Don't use routine drains, nasogastric tubes or urinary catheters; Early refeeding; Early mobilization.	Length of hospital stay (days, mean)—11.5 (control) × 8 (intervention); 30‐day mortality rate (%)—3% (control) × 1% (intervention) Postoperative fistula (%)—6% (control) × 4% (intervention)	—
Mc Loughlin [27]	Preoperative education; No routine mechanical bowel preparation; Preoperative fasting abbreviation; Opioid sparing anesthesia; Minimally invasive surgical approach; Don't use routine drains, nasogastric tubes or urinary catheters; Restrictive intravenous fluids administration; Early mobilization;	Length of hospital stay (days, mean)—4 days; PONV on the 2 first PO days (%)—22%;	—
Nari [28]	Preoperative education; Preoperative fasting abbreviation; Restrictive intravenous fluids administration; Don't use routine drains, nasogastric tubes or urinary catheters; Opioid sparing anestesia; Early refeeding; Early mobilization;	Length of stay (median, days)—3.86 (open surgery) × 3.93 (laparoscopic surgery) Start of mobilization (median, hours)—12.79 h (open surgery) × 26,5 h (laparoscopic surgery) Complications (%)—14.3% (open surgery) × 13.3% (laparoscopic surgery)	—
Santiago [29]	Preoperative education; Preoperative fasting abbreviation; No routine mechanical bowel preparation; Opioid sparing anesthesia; Restrictive intravenous fluids administration; Don't use routine nasogastric tubes; Early refeeding; Early mobilization; Audit	Length of hospital stay (median, days)—4.9 days; Reoperation (%)—4.7% Fistula (%)—7.8%	—
Marquini [30]	Preoperative fasting abbreviation	Incidence of nausea—17.5% (Fresubin) × 20.5% (Juice) Incidence of vomiting—10% (Fresubin) × 11.7% (Juice) There were no anesthetic complications;	—
Marquini [31]	Preoperative fasting abbreviation.	There was no statistical difference in length of stay and incidence of infections between the juice and Fresubin groups;	The authors believe that the intervention did not influence hospitalization time or hospital costs probably because the hospital routinely performs medium—complexity care, with high demand and turnover.
de Carvalho [32]	Preoperative fasting abbreviation.	Length of stay (days, median)—4 days (Carbohydrate) × 5 days (Carbohydrate and protein); Postoperative complications (%)—81.82% (Carbohydrate) × 18.18% (Carbohydrate and protein); Death (%)—66.67% (Carbohydrate) × 33.33% (Carbohydrate and protein).	—
Checa [33]	Preoperative education; Respiratory physiotherapy; No routine mechanical bowel preparation; Restrictive intravenous fluids administrations; Preoperative fasting abbreviation; Opioid sparing anestesia; Minimally invasive surgical approach; Don't use routine drains and nasogastric tubes; Early refeeding; Early mobilization; Audit;	Length of hospital stay (median, days)—13.86 days (control) × 10.62 (ERAS/Open) × 6.86 (ERAS/Laparoscopic) Postoperative vomiting (%)—5.4% (control) × 14.5% (ERAS/Open) × 10.6% (ERAS/Laparoscopic) Reoperation (%)—6.2% (control) × 6.7% (ERAS/Open) × 0 (ERAS/Laparoscopic) Death (%)—3.3% (control) × 3.6% (ERAS/Open) × 0 (ERAS/Laparoscopic)	—
Neville [34]	Minimally invasive surgical approach; Opioid sparing anesthesia; Early refeeding; Early urinary catheters removed;	Length of hospital stay (days, median)—5 days (control) × 3 days (intervention); Reoperations (%)—0 (control) × 8.8% (intervention); Death (%)—3.1% (control) × 6.1% (intervention);	Costs of hospitalization (median, US$)—2765 US$ (control) × 2135 US$ (intervention); Overall costs until 30th POD (median, US$)—3563 US$ × 2511 US$.
Zandomenico [35]	Preoperative education; Preoperative fasting abbreviation; Opioid sparing anestesia; Avoid the uso of nasogastric tubes; Early refeeding;	They measured compliance with the ERAS protocols among bariatric surgery patients; Immediate complications (%)—57.3%; Death (%)—0.6%;	—
Núñez‐Venzor [36]	Preoperative education; Opioid sparing anestesia; Avoid urinary catheter; Restrictive intravenous fluids administration; No routine nasogastric tubes or drains; Early refeeding; Early mobilization;	Total hospital length of stay (mean, hours)—63,8h (Intervention) × 95,36h (Control) First 12h mild pain (VAS, %)—36.8% (Intervention) × 15.8% (Control) First 12h moderate‐severe pain (VAS, %)—63.2% (Intervention) × 84.2% (Control) Reoperations (%)—15.8% (Intervention) × 5.3% (Control)	—
Oliveira [37]	Preoperative education; Early mobilization; Opioid sparing anesthesia; Rehabilitation.	They described the edema reduction measures	—
Almeida [38]	Preoperative education; Opioid free protocol; Avoid urinary catheterization; Early mobilization;	Length of hospital stay up to 6 days (%)—43.1% (Control) × 100% (Intervention); Complications (%)—3.9% (Control) × 10.6% (Intervention)	—
Oliveira [39]	Preoperative fasting abbreviation; Avoid urinary catheterization; Opioid sparing anesthesia; No routine nasogastric tubes or drains; Restrictive intravenous fluids administration; Early refeeding; Early mobilization;	Length of hospital stay (days, mean)—3.02 days (Control) × 2.04 days (Intervention); Complications (%)—25% (Control) × 11.5% (Intervention); Reoperations (%)—1.8% (Control) × 0 (Intervention)	Total hospitalization costs (US$, mean ± SD)—4506.42 ± 1172.53 (Control) x 3821.58 ± 1104.44 (Intervention). An analysis of variance (ANOVA) showed that postoperative discharge time was the main factor reducing hospitalization costs (*p* < 0.05).
Mejia [40]	Preoperative education; Preoperative use of pregabalin; Preoperative fasting abbreviation; Opioid sparing anesthesia and protective ventilation strategies; Early extubation, refeeding and mobilization; Early drain removal; Postoperative education and counseling;	Postoperative stay ≤ 5 days (%)—87%; Extubation ≤ 2 h (%)—39%	—
Martínez‐Mardones [41]	Preoperative education; Preoperative fasting abbreviation; Opioid sparing anesthesia; Restrictive intravenous fluids administration; No routine nasogastric tubes or drains; Early refeeding; Early mobilization; Audit.	Length of hospital stay (median, days)—4 days; Complications, Clavien‐Dindo I and II (%)—25%; They measured compliance with the ERAS protocols;	—
Nari [42]	Preoperative education; Preoperative fasting abbreviation; Restrictive intravenous fluids administration; No routine nasogastric tubes or drains; Opioid sparing anesthesia; Early refeeding; Early mobilization;	Length of hospital stay (mean, days)—3.9 days; Reoperation (%)—1.25%; Complications (%)—16.25%; Readmission (%)—5%.	—
Aguilar‐Nascimento [43]	Preoperative education; Preoperative fasting abbreviation; 5 days of oral protein supplements; Early refeeding; Restrictive intravenous fluids administration; No mechanical bowel preparation; No routine nasogastric tubes or drains; Early mobilization;	Length of Stay (Median, interquartile range)—13/12 (Control) × 10/12 (Intervention) Surgical Site Infection (%)—20.9% (Control) × 9.2% (Intervention) Postoperative complications (%)—33%.9% (Control) × 19% (Intervention) Mortality (%)—10% (Control) × 5.6% (Intervention).	Mean cost in Brazilian Reais per patient (R$)—24562,00 R$ (Control) x 19912,00 R$ (Intervention). Mean reduction was R$4650.03.
Stahlschmidt [44]	Preoperative education; Early PACU discharge; Rehabilitation.	—	—
Herros‐García [45]	Preoperative education; Respiratory physiotherapy; Preoperative fasting abbreviation; Opioid free anestesia; Early mobilization; Rehabilitation.	Pain 2 months after surgery (VAS, mean ± SD)—3.4 ± 1.3 (Intervention) × 4.2 ± 1.4 (Control)	—
Mendoza‐Vélez [46]	Preoperative education; Opioid sparing anesthesia; Restrictive intravenous fluids administration; Minimally invasive surgical approach; No routine nasogastric tubes or drains; Early mobilization; Early refeeding;	Postoperative pain (VAS, mean)—4.1; Length of hospital stay (hours, mean)—4.6 h; Postoperative complications (%)—3.1%.	—
Gruber [47]	Preoperative education; Preoperative fasting abbreviation; Restrictive intravenous fluids administration; Opioid sparing anesthesia; Early mobilization; Early refeeding;	Complications (%)—29.3%; Pain (%)—15.2%; PONV (%)—11.1%; Readmission (%)—20.2%; Length of stay (mean, hours)—11.52 h.	—
Marra [48]	Preoperative education;	Educational intervention—They measured the adherence and knowledge of ACERTO protocol.	—
Pineyro [49]	No mechanical bowel preparation; Preoperative fasting abbreviation; Opioid sparing anesthesia; Restrictive intravenous fluids administration; Early refeeding; Early ambulation;	Postoperative pain on the first 24 h (VAS, mean ± SD)—2.8 ± 2.1 (Control) × 4.0 ± 1.3 (Intervention) Postoperative hospital stay (hours, mean ± SD)—44.0 ± 5.4 (Control) × 50.2 ± 8.2 (Intervention); Complications (%)—2.1% (Control) × 2.6% (Intervention)	—
Fernandez‐Portilla [50]	No mechanical bowel preparation;	Length of hospital stay (days, median)—4 (control) × 4 (intervention)	—
Fonseca [51]	Preoperative education; Opioid sparing anesthesia; Goal‐directed fluid therapy; Avoid the use of abdominal drain; Early removal of nasogastric tube and urinary catheter; Early mobilization; Early refeeding; Respiratory and motor physiotherapy.	Length of hospital stay (days, mean)—8.4 days (Control) × 5.1 days (Intervention); Complications (%)—53% (Control) × 30% (Intervention); Reoperation (%)—8% (Control) × 3% (Intervention).	—
Frizon [52]	Early refeeding;	Delayed postoperative refeeding and the use of an intra‐abdominal drain (*p* = 0.007) independently increase the risk of hospital stays longer than 5 days.	——
Lima [53]	Preoperative education; Preoperative fasting abbreviation; Opioid sparing anesthesia; Restrictive intravenous fluids administration; Early mobilization; Early refeeding; Audit.	Length of hospital stay (days, mean)—2.8 days;	—
Riquoir [54]	Early mobilization; Preoperative fasting abbreviation; Early refeeding;	Length of hospital stay (days, median)—3 days (Control) × 3 days (Intervention); 30‐day readmission (%)—2.53% (Control) × 0 (Intervention)	—
Arruda [55]	Preoperative fasting abbreviation;	Length of hospital stay (days, mean)—7.5 days (Control) × 2.24 days (Intervention);	Mean costs ± SD in Brazilian Reais per patient (R$)—6193.83 ± 11414,72 R$ (Control) x 3284.32 ± 4570.84 R$ (Intervention)
Gonçalves [56]	Preoperative education; Preoperative fasting abbreviation; Audit	They measured factors associated with extended fasting times	—
Hernandez‐Gaytán [57]	Preoperative education; Preoperative fasting abbreviation; Opioid sparing anesthesia; Restrictive intravenous fluids administration; Early mobilization;	24 h Postoperative pain (VAS, mean ± SD)—4.1 ± 2.01 (Control) × 2.65 ± 2.3 (Intervention) Length of stay (days, mean ± SD)—3.39 ± 1.85 (Control) × 1.59 ± 0.70 (Intervention).	—
Magro [58]	Preoperative education; Preoperative fasting abbreviation; Early refeeding;	They measured factors associated with perioperative nutritional support.	—

The most studied enhanced recovery strategies were: Preoperative fasting abbreviation with 26 studies, early refeeding and early mobilization appears in 22 studies each, opioid‐sparing anesthesia with 19 studies, preoperative education and restrictive intravenous fluids administration was studied in 16 studies each.

Regarding the economic impact and the hospitalization costs, 34 studies (87.2%) did not report any data about the topic. Only five [31, 34, 39, 43, 55] studies provided cost‐related findings. While five studies reported direct microeconomic measures, such as hospitalization costs (R$ or US$), the analysis identified a complete absence of data pertaining to the macroeconomic impact of ERAS protocols, including metrics that quantify benefits beyond the hospital setting, such as return‐to‐work time or productivity gains.

## 4 Discussion

### 4.1 Synthesis of Patient Outcomes Associated With ERAS

This scoping review demonstrates a substantial body of evidence addressing the implementation of Enhanced Recovery After Surgery (ERAS) protocols in Latin America and the Caribbean, with a total of 45 studies included. These studies encompassed a wide range of clinical outcomes, reflecting the multidimensional impact of ERAS pathways in surgical care. The most frequently evaluated outcomes were length of hospital stay, postoperative complications (infection, ileus, pneumonia, atelectasis and Clavien–Dindo grade I–II events), postoperative pain, postoperative nausea and vomiting, and early refeeding.

In addition to these commonly reported outcomes, several studies explored less frequently assessed endpoints. These included the evaluation of inflammatory markers, reoperations, mortality, hospital readmission, and adherence to ERAS processes, highlighting the heterogeneity of outcome selection and the relative scarcity of data on system‐level and long‐term measures. This section synthesizes the patient outcomes most frequently reported in ERAS studies from Latin America and the Caribbean, with individual paragraphs dedicated to each major clinical outcome.

Length of hospital stay was evaluated in 36 studies and represented the most frequently reported clinical outcome. A statistically significant reduction in postoperative hospitalization following ERAS implementation was demonstrated in 21 studies [14, 18, 19, 20, 21, 23, 24, 25, 26, 27, 33, 34, 36, 38, 39, 40, 43, 49, 50, 51, 57]. An additional 13 studies reported a reduction in length of stay without statistical analysis or without reaching statistical significance [15, 16, 22, 28, 29, 31, 32, 41, 42, 46, 47, 53, 55]. No reduction in hospital stay was observed in two studies, one without statistical significance [17] and one showing a statistically significant absence of reduction [54]. Overall, these findings indicate that shortened length of stay is a frequent and reproducible outcome associated with ERAS implementation, with neutral findings occurring predominantly in studies with limited statistical power or descriptive designs.

Postoperative complications were evaluated in 20 studies [14, 15, 21, 24, 25, 26, 27, 28, 29, 32, 35, 38, 39, 41, 42, 43, 46, 47, 49, 51]. The most frequently reported events were surgical site infections [14, 15, 21, 24, 25, 26, 28, 29, 32, 42, 43, 46, 49, 51] in 14 studies, followed by anastomotic fistula or wound dehiscence [14, 15, 21, 26, 29, 35, 39, 42, 43, 51] analyzed by 10 studies, postoperative ileus [21, 27, 41, 42] in 4 studies, and pulmonary complications [24, 35, 42, 47] in 4 studies. A statistically significant reduction in postoperative morbidity after ERAS implementation was observed in eight studies [14, 21, 24, 27, 32, 39, 43, 51], while 10 studies reported reductions without statistical analysis or without reaching statistical significance [15, 26, 28, 29, 35, 38, 41, 46, 47, 49]. Two studies found no reduction in complication rates [25, 42]. Overall, ERAS protocols showed a favorable safety profile, although heterogeneity in outcome definitions limits direct cross‐study comparisons.

Postoperative pain was evaluated in nine studies [15, 17, 20, 36, 45, 46, 47, 49, 57]. A statistically significant reduction in postoperative pain and/or opioid consumption following ERAS implementation was reported in three studies [20, 49, 57], while five studies described reductions without statistical analysis or without reaching statistical significance [15, 17, 45, 46, 47]. One study found no reduction in postoperative pain [36]. Overall, ERAS protocols were associated with effective postoperative analgesia, although heterogeneity in pain assessment methods limits direct cross‐study comparisons.

Postoperative nausea and vomiting was evaluated in seven studies [17, 18, 25, 27, 30, 33, 47]. A statistically significant reduction in PONV following ERAS implementation was reported in two studies [25, 27], while five studies described reductions without statistical analysis or without reaching statistical significance [17, 18, 30, 33, 47]. Overall, ERAS protocols were associated with lower rates of PONV without evidence of harm, although heterogeneity in outcome definitions and assessment methods limits direct cross‐study comparisons. Taken together, the evidence across length of hospital stay, postoperative complications, pain, and PONV indicates that the principal clinical outcomes relevant to perioperative recovery were consistently assessed and generally favored ERAS implementation in Latin America and the Caribbean.

### 4.2 Geographic and Structural Disparities in ERAS Implementation

Despite a relatively large number of identified studies, the regional distribution of optimizing surgical strategies research remains highly uneven. The preponderance of evidence originates from Brazil, Mexico, Argentina, and Chile, with Brazil alone contributing over half of all included studies. Conversely, the vast majority of Latin American and Caribbean nations, including Central America and the Caribbean islands, lack any published ERAS research. This geographic imbalance is consistent with structural disparities in research capacity, funding, and surgical infrastructure. It also suggests that implementation may be concentrated in higher‐resource or academically oriented centers, potentially limiting the extrapolation of current findings across the region's diverse health systems.

This geographic concentration closely parallels regional patterns of health‐system financing and organization described by Atun et al. [59] Countries such as Brazil, Argentina, Chile, and Mexico (those contributing most ERAS‐related publications) also demonstrate higher health expenditure per capita and a greater proportion of public financing, reflecting more structured and coordinated health systems. In contrast, regions with lower levels of investment and more fragmented health financing, particularly Central America and the Caribbean, remain largely absent from the ERAS literature. Estimates from the Global Surgery 2030 Commission further highlight substantial unmet surgical need across Latin America, particularly in tropical and Central Latin American regions [60].

In this context, the limited diffusion of ERAS protocols to under‐resourced settings represents a potential missed opportunity for health‐system efficiency. ERAS pathways are designed to optimize perioperative resource utilization and have been associated with reductions in length of hospital stay and hospital‐level costs, outcomes that may be particularly relevant for health systems facing fiscal constraints. The current concentration of ERAS evidence in a small number of countries may therefore limit the representativeness of available data and underscores the importance of evaluating ERAS implementation as a cost‐conscious, system‐strengthening intervention across diverse healthcare environments in Latin America and the Caribbean. Addressing these geographic and structural disparities is essential to support more equitable dissemination of ERAS protocols and to maximize their potential clinical and economic benefits at a regional level.

### 4.3 Lessons From International Comparisons

These disparities parallel the findings of the Latin American Surgical Outcomes Study (LASOS) [9], which showed that although perioperative complications occurred at rates comparable to high‐income countries, mortality following complications was significantly higher in Latin America. The LASOS results highlight gaps in postoperative monitoring, access to intensive care, and the organization of perioperative pathways: factors directly addressed by ERAS programs. The overlap between the countries with the most ERAS research and those with better‐performing hospitals in LASOS reinforces the idea that ERAS implementation tends to emerge where health systems already exhibit stronger perioperative performance. Conversely, regions with poorer infrastructure (where ERAS could provide the greatest benefit) remain underrepresented in the literature.

International comparisons further contextualize these findings. A recent scoping review from Africa by Kifle et al. [7] reported challenges, including variability in guideline adherence, insufficient resources, and limited multidisciplinary engagement, despite growing ERAS interest. The parallels between Latin America and Africa underscore common systemic barriers in low‐middle‐income settings: fragmented perioperative care, personnel shortages, resistance to practice change, and insufficient institutional support. Crucially, these barriers are often rooted in deep socioeconomic inequity, resulting in stark disparities in access to high‐quality surgical care even within the same urban center. The African review also highlighted the transformative potential of ERAS when tailored to resource‐constrained environments, an approach Latin America could similarly leverage through regional capacity‐building strategies.

Experiences from high‐income countries indicate that ERAS benefits are achieved not through protocol adoption alone, but through progressive implementation, multidisciplinary engagement, and continuous audit and feedback. Large international studies have shown a direct association between higher adherence to ERAS components and improved clinical outcomes, whereas partial implementation yields attenuated benefits. These findings reinforce that ERAS represents a complex organizational intervention rather than a simple checklist and suggest that in low‐ and middle‐income settings such as Latin America, contextual adaptation and phased implementation focused on high‐impact, low‐cost components may optimize clinical and economic gains despite structural constraints.

### 4.4 Economic Impact of ERAS: What Is Known and What Remains Unclear

The economic evaluation of Enhanced surgical recovery implementation constitutes a major gap in the Latin American and Caribbean literature. Out of 45 studies identified, only five [31, 34, 39, 43, 45] reported cost‐related outcomes. These microeconomic evaluations consistently demonstrated reductions in hospitalization costs associated with ERAS protocols, primarily driven by shorter postoperative length of stay and fewer complications. For instance, Neville et al. [34] and Oliveira et al. [39] showed meaningful decreases in direct hospital expenditures, with Oliveira et al. [39] identifying discharge time as a key determinant of cost reduction. Conversely, all these analyses were limited strictly to hospital‐based costs, omitting broader macroeconomic benefits such as workforce productivity, patient return‐to‐work, or health‐system–level resource optimization. This scarcity of robust economic analyses underscores the critical need for future research to quantify the societal and system‐wide financial impact of ERAS in the region.

Importantly, the existing economic evidence is limited not only in quantity but also in scope and methodological depth. All identified studies adopted a hospital‐based microeconomic perspective, focusing on direct inpatient costs, without formal cost‐effectiveness, cost–utility, or budget impact analyses. None incorporated indirect costs borne by patients or society, such as productivity losses, caregiver burden, or system‐level effects related to bed turnover and surgical throughput. Moreover, heterogeneity in costing methods, currencies, and time horizons limits cross‐study comparability. Consequently, while available data suggest that ERAS implementation may reduce direct hospital expenditures, the true economic value of ERAS at the health‐system and societal levels in Latin America and the Caribbean remains largely undefined. Addressing these gaps through methodologically robust economic evaluations is essential to inform policy decisions, guide sustainable ERAS adoption, and ultimately maximize the clinical and economic benefits of enhanced recovery pathways across the region.

## 5 Conclusion

This scoping review demonstrates that ERAS initiatives in Latin America and the Caribbean are expanding, with consistent evidence of reduced hospital length of stay and signals of cost savings in the limited number of studies that assessed economic outcomes. However, ERAS implementation remains uneven, largely concentrated in Brazil, Mexico, Argentina, and Chile, while much of Central America and the Caribbean remains underrepresented. This geographic imbalance limits the generalizability of current findings and reflects broader structural disparities in perioperative care across the region.

While the existing literature provides substantial evidence on short‐term clinical outcomes, it offers limited insight into implementation processes, long‐term sustainability, and system‐level economic impact. In this context, prospective studies are needed not to reconfirm clinical efficacy, but to clarify how ERAS can be effectively implemented and sustained across diverse healthcare settings, to quantify its cost‐effectiveness and budget impact from health‐system and societal perspectives, and to identify context‐specific barriers and facilitators to adoption. Generating this evidence will be essential to support policy decisions, guide resource allocation, and establish ERAS as a scalable and economically justified standard of perioperative care throughout Latin America and the Caribbean.

## Author Contributions

**Lucas Ferreira Gomes Pereira:** conceptualization, data curation, formal analysis, investigation, methodology, project administration, resources, supervision, validation, visualization, writing – original draft, writing – review and editing. **José Eduardo Guimarães Pereira:** conceptualization, data curation, formal analysis, methodology, supervision, writing – review and editing. **Vinicius Caldeira Quintão:** conceptualization, formal analysis, methodology, project administration, resources, supervision, writing – review and editing. **Luiz Fernando dos Reis Falcão:** conceptualization, investigation, methodology, supervision, validation, writing – review and editing. **Bruno Araújo Borges:** conceptualization, investigation, validation, visualization, writing – original draft. **Bruce Biccard:** conceptualization, methodology, project administration, supervision, writing – review and editing. **Carlos Darcy Alves Bersot:** conceptualization, data curation, formal analysis, investigation, methodology, project administration, supervision, visualization, writing – review and editing.

## Funding

The authors have nothing to report.

## Conflicts of Interest

The authors declare no conflicts of interest.

## Data Availability

The data that support the findings of this study are openly available in Open Science Framework at https://osf.io/9x7by/.
